# From seizure to stability: unveiling the brain’s network changes with anti-seizure medication

**DOI:** 10.1093/braincomms/fcae335

**Published:** 2024-09-27

**Authors:** Mark J Cook

**Affiliations:** Faculty of Engineering and Information Technology, The University of Melbourne, Melbourne 3010, Victoria, Australia

## Abstract

This scientific commentary refers to ‘Brain network changes after the first seizure: an insight into medication response?’, by Pedersen *et al*. (https://doi.org/10.1093/braincomms/fcae328).

This scientific commentary refers to ‘Brain network changes after the first seizure: an insight into medication response?’, by Pedersen *et al*. (https://doi.org/10.1093/braincomms/fcae328).

Epilepsy presents a significant challenge for both clinicians and patients. The decision to initiate an anti-seizure medication (ASM) after a first epileptic seizure is complex, driven by the need to balance the prevention of further seizures with the potential risks associated with medication.^1^ The recent prospective study by Pedersen *et al*.^2^ in *Brain Communications*, which utilized advanced neuroimaging techniques, provides valuable insights into the brain network changes that occur following ASM monotherapy in individuals who have experienced a first seizure.

The study employed graph theoretical network analysis on longitudinal resting-state functional MRI (fMRI) data from 28 participants. Imaging was conducted both before and during long-term ASM therapy, with an average interval of 6.9 months between scans. The findings indicate significant changes in brain network organization after ASM administration, with particularly pronounced effects in participants who experienced recurrent seizures.

The study found that the clustering coefficient, a measure of local connectivity within brain networks, increased following ASM therapy. This increase suggests that ASM may promote the stabilization of neural circuits, potentially reducing the likelihood of further seizures.^3,4^ Enhanced local connectivity could reflect the brain’s attempt to reorganize itself in response to the abnormal electrical activity that characterizes epilepsy.^5^

A decrease in network path length, which indicates more efficient communication across the brain, was observed after ASM initiation. This suggests that ASM may help to restore or improve the overall efficiency of brain networks, potentially counteracting the disruptive effects of seizure activity.^4^ More efficient networks are typically associated with better cognitive functioning, which could have implications for the cognitive outcomes of patients undergoing ASM therapy.^6^

The most significant changes in brain network metrics were observed in the superior frontoparietal and inferior frontotemporal regions. These areas are known to be involved in critical cognitive functions, such as attention, executive function, and memory, which are often affected in individuals with epilepsy,^7^ though there are questions as to whether seizures or medications themselves are making the greatest contribution to the situation. The regional specificity of these changes highlights the targeted effects of ASM on brain networks, particularly in regions that may be most vulnerable to the impact of seizures.^8^

Participants who experienced recurrent seizures showed the most pronounced changes in brain network organization after ASM treatment. This finding suggests that the brain’s response to ASM may be influenced by the frequency and severity of seizure activity.^9^ It is possible that recurrent seizures lead to more extensive network disruption, which ASM then attempts to mitigate. Alternatively, the pronounced changes observed in these participants could reflect an ongoing process of network reorganization as the brain adapts to both seizure activity and ASM therapy.^10^

The findings of this study have important implications for the clinical management of epilepsy, particularly in the context of initiating ASM after a first seizure. The absence of clinically useful biomarkers in epilepsy management is a major impediment currently to identifying effective treatment. The observed changes in brain network properties could serve as potential biomarkers for monitoring the effects of ASM and predicting treatment outcomes.^10^ If these changes are consistently associated with a reduced likelihood of further seizures, they could inform decisions about when to initiate or adjust ASM therapy.

Additionally, the study underscores the broader impact of ASM on brain function beyond seizure control. The alterations in brain network organization observed after ASM initiation suggest that these medications may have cognitive and behavioural effects that need to be carefully monitored.^11^ Clinicians should be aware of these potential effects and consider them when making treatment decisions, particularly in patients who are at risk of cognitive decline or other neurological impairments,^11^ and this potentially provides an insight into how to identify those at risk.

While the study provides valuable insights into the effects of ASM on brain networks, several limitations must be acknowledged. The small sample size limits the generalizability of the findings, and larger studies are needed to confirm these results.^4^ Additionally, the lack of a control group that did not receive ASM makes it difficult to determine whether the observed changes are due to the medication itself or other factors, such as the natural course of epilepsy or changes in lifestyle and behaviour after a seizure.^3^

Future research should aim to include control cohorts and explore the effects of different types of ASM on brain networks. Additionally, incorporating task-based fMRI and other neuroimaging techniques could provide a more comprehensive understanding of how ASM affects both resting-state and task-related brain activity.^6^ Longitudinal studies that follow patients with a variety of underlying pathologies and varying seizure frequencies over extended periods are also needed to assess the long-term effects of ASM on brain network organization and their relationship to seizure outcomes.^10^

This study represents an important step forward in our understanding of how ASM therapy affects brain network function after a first epileptic seizure. The observed changes in clustering coefficient and network path length, particularly in participants with recurrent seizures, suggest that ASM has a significant impact on brain network organization.^8^ These findings have the potential to guide clinical practice by providing new biomarkers for monitoring treatment effects and guiding personalized therapy in epilepsy.

Further research is needed to fully elucidate the relationship between ASM-induced brain network changes and long-term seizure outcomes. Larger, controlled studies incorporating diverse neuroimaging techniques and clinical endpoints will be essential to translate these findings into clinical practice and improve the management of epilepsy.^9^

This study marks the first steps towards disentangling the complex relationship between brain network dynamics and ASM, offering a glimpse into the future of personalized epilepsy care. The observed changes in neural connectivity not only underscore the profound impact of ASM on brain function but also illuminate the potential for developing reliable biomarkers that could revolutionize how we select and monitor epilepsy treatments. These findings, though preliminary, lay the groundwork for a deeper understanding of the brain’s adaptive mechanisms in response to therapeutic interventions. As we continue to refine these insights, we edge closer to a paradigm where epilepsy management is guided by precise, individualized biomarkers, ensuring that each patient receives the most effective and tailored care. This journey is just beginning, but it holds the promise of transforming the landscape of epilepsy treatment and improving outcomes for countless individuals living with this challenging condition.

## Data Availability

Not applicable.
